# Maternal microbiome in preeclampsia pathophysiology and implications on offspring health

**DOI:** 10.14814/phy2.14875

**Published:** 2021-05-27

**Authors:** Jeanne A. Ishimwe

**Affiliations:** ^1^ Department of Pharmacology and Toxicology University of Mississippi Medical Center Jackson MS USA

**Keywords:** developmental programming, dysbiosis, gut, microbiome, periodontal, placenta, preeclampsia, vagina

## Abstract

Preeclampsia is a devastating hypertensive pregnancy disorder that currently affects 2%–8% of pregnancies worldwide. It is associated with maternal and fetal mortality and morbidity and adverse health outcomes both in mom and offspring beyond pregnancy. The pathophysiology is not completely understood, and there are no approved therapies to specifically treat for the disease, with only few therapies approved to manage symptoms. Recent advances suggest that aberrations in the composition of the microbiome may play a role in the pathogenesis of various diseases including preeclampsia. The maternal and uteroplacental environments greatly influence the long‐term health outcomes of the offspring through developmental programming mechanisms. The current review summarizes recent developments on the role of the microbiome in adverse pregnancy outcomes with a focus on preeclampsia. It also discusses the potential role of the maternal microbiome in fetal programming; explores gut‐targeted therapeutics advancement and their implications in the treatment of preeclampsia.

## Graphical abstract

Preeclampsia is a devastating hypertensive pregnancy disorder with maternal and fetal mortality and morbidity, and the maternal and uteroplacental environments greatly influence the long‐term health outcomes of the mom and offspring. Recent advances suggest that aberrations in the composition of the microbiome may play a role in the pathogenesis of preeclampsia. The current review summarizes recent developments on the role of the microbiome in adverse pregnancy outcomes with a focus on preeclampsia and explores gut‐targeted therapeutics advancement and their implications in the treatment of preeclampsia.

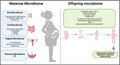

## 1 INTRODUCTION

Preeclampsia is a devastating multifactorial pregnancy disorder that currently affects 2%–8% of pregnancies worldwide. Previously, preeclampsia was defined by the new‐onset of hypertension around 20 weeks of gestation and proteinuria (American College of Obstetricians & Gynecologists; Task Force, 2013). Recent advances now acknowledge the disease by new‐onset hypertension in pregnancy (systolic blood pressure ≥140 mmHg and diastolic blood pressure ≥90 mmHg) and damage to one or more other organ systems presenting through the following features: proteinuria (≥300 mg/ 24 h) or protein (mg/dl)/creatinine ratio (mg/dl) ≥0.3, hemolysis, elevated liver enzymes and low platelet count (<100,000/μl), neurological or visual symptoms, abnormal Doppler ultrasound, and fetal growth restriction (Brown et al., 2018). Preeclampsia is associated with maternal and fetal mortality and morbidity causing 76,000 maternal and 500,000 fetal deaths each year globally (American College of Obstetricians & Gynecologists; Task Force, 2013). Additionally, it poses a great financial burden where healthcare costs for preeclamptic pregnancies for both mothers and infants are estimated at $2.18 billion for the first 12 months after delivery in the United States alone (Stevens et al., 2017).

Preeclampsia is classified as early‐onset if diagnosed before 34 weeks of gestation and late‐onset if diagnosed after 34 weeks (Redman & Sargent, 2001; Roberts & Bell, 2013). Although, the origins and severity of adverse outcomes associated with each subtype may vary, the overall pathophysiology is still not completely understood. There remain no approved therapies to specifically treat the disease, and only a few therapies are approved to manage symptoms. As of yet, there are no diagnostic biomarkers that can accurately predict who will get preeclampsia before pregnancy, but there are factors and comorbidities that are known to increase a woman's risk for developing the disease including advanced maternal age (≥35 years), inadequate nutrition, chronic hypertension, obesity, and diabetes (Alexander, 2006; Delhaes et al., 2018; Pinheiro et al., 2019; Poon et al., 2019; Seely & Ecker, 2014). The incidence of some of these factors is steadily rising, further raising concerns that the prevalence of preeclampsia may also increase in the years to come (Cho et al., 2018; Finkelstein et al., 2012).

Preeclampsia is clinically diagnosed after 20 weeks of gestation, but clinical and experimental findings suggest the disease initiating events may start during the implantation/placentation period. Defective placentation through impairments in the spiral arteries remodeling process and the persistent placental ischemic insult that ensues are believed to be among the initiating factors in the pathophysiology of preeclampsia (Phipps et al., 2019). While placental‐derived molecules are required for a successful pregnancy, dysregulation in the pathways involved has been implicated in pregnancy disorders. Our understanding of the exact mechanisms that cause preeclampsia remains incomplete. Current evidence shows that in response to placental hypoxia, the placenta releases various factors into the maternal circulation that may contribute to the disease development, and there may be an imbalance between proangiogenic and antiangiogenic factors. Vascular endothelial growth factor (VEGF) is a proangiogenic factor that primarily regulates angiogenesis through the VEGF receptor‐1 (VEGFR‐1) and fms‐like tyrosine kinase‐1 (Flt‐1). In preeclampsia, a soluble form of Flt‐1, sFlt‐1, is elevated in circulation and binds to VEGF, antagonizing its proangiogenic role (Levine et al., 2004; Maynard et al., 2003). As a result, free VEGF is significantly lower in pregnancies complicated with preeclampsia compared to normal pregnancy (Umapathy et al., 2020). Circulating levels of placental growth factor (PlGF), another proangiogenic protein, are decreased in preeclampsia. Soluble endoglin is an anti‐angiogenic molecule that exacerbates vascular dysfunction by decreasing endothelial nitric oxide signaling and is increased in preeclampsia (James et al., 2010). Oxidative stress from the maladapted placental blood flow and dysregulated non‐specific inflammatory responses contribute to a heightened maternal global pro‐inflammatory state (Redman & Sargent, 2010). Great efforts have been made in identifying predictive markers for the development of preeclampsia. Circulating sFlt‐1, PlGF, and sFlt‐1:PlGF ratio have been reported to be potentially useful in predicting women who are at risk so they can be monitored closely following clinical guidelines (Agrawal et al., 2019; Levine et al., 2004; Souders et al., 2015; Verlohren et al., 2012; Widmer et al., 2015). Clearly, there is still a dire need to continue elucidating the mechanisms and develop treatments for preeclampsia. The purpose of this review is to summarize the key recent advances in our understanding on the role of the microbiome in preeclampsia. We highlight mechanistic links between mechanisms of the oral, gut, vaginal, and placenta microbiomes and the etiology of preeclampsia. We review current preclinical models of preeclampsia and postulate on future research areas that these models may be useful for. Lastly, we review currently available microbiome therapeutics and potential future treatment avenues for preeclampsia that target the microbiome.

## 2 MICROBIOME AND HOST HEALTH

Microbiome, composed of trillions of microbes and their collective genetic material, is now an established contributor to normal physiology and disease (Ding et al., 2019; Kashyap et al., 2017). Microbes have been studied for their pathogenic role, mainly in infectious diseases, for centuries, and the existence of bacteria in the gut has been known for over 100 years (Aziz, 2009). Recently, there has been a paradigm shift in the field of microbiome and all the many ways it contributes to health and disease, particularly recognizing the connection and axes between microbiome environments like the gut and other organs (Carding et al., 2015; Mohajeri et al., 2018; Wilck et al., 2017; Yang et al., 2019). In 2007, the National Institutes of Health launched the Human Microbiome Project to characterize the microbiome composition at various body sites (Lloyd‐Price et al., 2016). So far, the most studied microbiota in humans are oral, skin, urogenital, and gastrointestinal microbiome. There is no definition of a healthy human microbiome based on the high prevalence or lack thereof of certain taxa due to the highly dynamic nature of the microbiome (Lloyd‐Price et al., 2017; Yatsunenko et al., 2012). The proposed alternative hypothesis is to characterize a healthy microbiome based on functional microbiome core (Lloyd‐Price et al., 2016). This is because unlike microbial relative abundance, their metabolic pathways are reported to be established early in life and to remain stable in the absence of disease (Kostic et al., 2015).

Recent advances suggest that disruption in the composition of the microbiome may play a role in the pathogenesis of various diseases. There is mounting evidence supporting the involvement of the microbiome in cardiovascular health‐related disorders that are also known to be risk factors for preeclampsia including obesity and diabetes (Crusell et al., 2018, 2020; Halkjaer et al., 2016; Xue et al., 2014). Of the microbiomes, the gut bacterial microbiota has been the most studied and best characterized. Over the last 5 years, numerous reports both in humans and animal models have shown an association between alterations in the gut microbiome and hypertension (Kim et al., 2018; Kim, Rigatto, et al., 2020; Li et al., 2017; Mell et al., 2015; Santisteban et al., 2017; Wilck et al., 2017; Yan et al., 2017; Yang et al., 2016). There is also direct evidence whereby fecal microbiota transplantation from a hypertensive individual or animal elicits hypertension in a previously normotensive rodent (Toral et al., 2019). The role of other microbes such as viruses and fungi is much less understood even though they are well known to be pathobionts in various infectious and inflammatory diseases. With recent methodological advancements, emerging reports are broadening our understanding of the microbiome by recognizing the potential contribution of other microbes other than bacteria including the virome and mycobiome (Azevedo et al., 2020; Li, Li, et al., 2019; Maqsood et al., 2019; Scarpellini et al., 2015; Vemuri et al., 2020). For example, the gut virome is altered in hypertensive patients and differentiated between normotensive and hypertensive phenotypes better than gut bacteria (Han et al., 2018).

## 3 MICROBIOME IN PREECLAMPSIA

Normal pregnancy is associated with numerous hormonal, metabolic, and immunological changes that are associated with other diseases outside of pregnancy but are healthy adaptation to pregnancy (Newbern & Freemark, 2011). Thus, before understanding what governs disease‐promoting dysbiosis in preeclampsia, it is imperative that we first identify gut microbiome changes in normal pregnancy. Studies exploring the role of the microbiome in preeclampsia need to investigate which changes occur during which phase of pregnancy to better distinguish adaptive from maladaptive changes as well as their onset. A longitudinal study exploring the gut microbiome demonstrated that the alterations vary by pregnancy stages. Alpha or within‐subject diversity decreases whereas beta or between‐subject diversity expands. The microbiome at the beginning of a healthy pregnancy is “healthy” whereas it resembles a disease‐promoting dysbiosis towards the end of gestation. For example, Proteobacteria and Actinobacteria phyla are increased by late pregnancy (Koren et al., 2012) and the phyla have been identified as pathogenic in disorders outside of pregnancy. Evidence supporting the involvement of the microbiome in preeclampsia are also emerging. Four microbial sites have been implicated to potentially contribute to the disease etiology: oral, gut, placental, and vaginal microbiome. It also remains unclear whether there is an interplay of multiple microbiomes as studies looking at more than one microbiome environment are lacking. A study evaluating longitudinal variation the human microbiota during pregnant identified vaginal microbiome alterations to be more related to preterm delivery compared to the gut and oral microbiomes (DiGiulio et al., 2015). Below, we review current literature on each of the four microbial sites and their known contribution to adverse pregnancy outcomes such as preeclampsia.

## 4 ORAL MICROBIOME

Oral health is recognized for its ability to impact host physiology. Periodontal pathogens are widely associated with preterm birth and fetal growth restriction (Jarjoura et al., 2005; Madianos et al., 2001). For example, *Porphyromonas gingivalis* infection causes fetal growth restriction and increased lethality (Collins et al., 1994). Treating periodontal disease in pregnant women significantly attenuates fetal growth restriction (López et al., 2002). The oral microbiome is also suggested to play a role in pregnancy disorders whose infection is not the primary cause. A longitudinal study conducted in pregnant women with gestational diabetes showed that oral microbial diversity is decreased in late pregnancy compared to healthy pregnant controls (Crusell et al., 2020). Women with gestational hypertension have higher periodontal pathogens compared to normotensive pregnant controls (Swati et al., 2012). Nitric oxide (NO) is an established regulator of blood pressure and targeting related pathways to increase NO production has been shown to mitigate preeclampsia (American College of Obstetricians & Gynecologists; Task Force, 2013; Gillis et al., 2016; Terstappen et al., 2019) Among microbes in the mouth is nitrate‐and nitrite‐reducing bacteria suggested to play a role in modulating blood pressure because mammals do not have effective nitrate reductase enzymes (Bryan et al., 2017; Hyde et al., 2014). Dietary inorganic nitrate supplementation in humans altered the oral microbiome, increased plasma nitrite levels which were associated with reduced blood pressure (Vanhatalo et al., 2018). Interestingly, a clinical trial in chronically hypertensive pregnant women showed an association between increased nitrate intake via beetroot juice and a reduction in blood pressure (Ormesher et al., 2018). Therefore, there is evidence both from human studies and animal models to implicate oral microbiome in mediating characteristics of preeclampsia‐like blood pressure and growth restriction. This not only further supports mechanisms such as NO bioavailability as a therapeutic approach for preeclampsia but also suggests a new avenue through these nitrate‐nitrite reducing bacteria.

## 5 GUT MICROBIOME

The most studied cavity thus far in cardiovascular health is the gut especially since it harbors the majority of the microbes and home to the largest immune organ, the gut‐associated lymphoid tissue (Shi & Gewirtz, 2018). The health of the gut microbiome can be assessed through its biodiversity; relative abundances of specific taxa; metabolic features; and the gut wall integrity (Ruan et al., 2020). Preeclampsia is associated with a disrupted gut microbiome that extends through 6 weeks postpartum (Lv et al., 2019). Studies have shown that in preeclampsia, bacterial diversity is different from that of normal pregnant women. Biodiversity is reported not to change in preeclampsia (Wang et al., 2020). However, preeclampsia is associated with increased Proteobacteria, Bacteroidetes, and Actinobacteria phyla compared to normal pregnancy controls. Au contraire, there is a significant reduction in Firmicutes and differential relative abundance in various genera. There are also reports of changes in metabolites associated with the gut microbiome during preeclampsia. Plasma lipopolysaccharide (LPS) and Trimethylamine‐N‐Oxide (TMAO) are increased in preeclamptic women (Chang et al., 2020; Wang et al., 2019). Interestingly, a recent study demonstrated that plasma levels of TMAO in pregnant women vary depending on the trimester and only late pregnancy levels were significantly associated with a risk for developing preeclampsia further highlighting the need for longitudinal studies (Huang et al., 2020). Most available evidence on the involvement of the gut microbiome in modulating pregnancy outcomes is from gut‐targeted interventional studies. Some studies show that prebiotics and probiotics may be safe and useful interventions, whereas others report no benefit (Dotterud et al., 2015; Grev et al., 2018; Okesene‐Gafa et al., 2019; Sohn & Underwood, 2017). We speculate that these discrepancies may be due to varying formulations as well as the pregnancy stage at which they were initiated.

## 6 VAGINAL MICROBIOME

The normal vaginal microbiota is composed of low diversity microbial communities reportedly dominated by *Lactobacillus* species. It undergoes transient changes in response to physiological and environmental factors such as hormones and hygiene (Al‐Nasiry et al., 2020). In a normal pregnancy, the vaginal microbiome is characterized by change in the relative abundance of *Lactobacillus* species and increased within‐subject diversity (MacIntyre et al., 2015). Others have shown that normal pregnancy is associated with changes in the relative abundance of these species with no difference between preterm and term delivery (Romero et al., 2014). Changes in the vaginal microbiome structure is associated with preterm birth (Hočevar et al., 2019; Petricevic et al., 2014). For example, depletion in certain vaginal lactobacilli species can result in conditions such as bacterial vaginosis. Perinatal treatment of bacterial vaginosis extends gestation and rescues fetal growth restriction (Kirihara et al., 2018). Also, preterm premature rupture of membranes, which often precedes preterm delivery, is associated with increased vaginal microbial diversity and reduction in *Lactobacillus* species suggesting their potential role as early risk factors for preterm delivery (Brown et al., 2019; Jayaprakash et al., 2016). Interestingly, a recent trial assessed the effect of an oral probiotic on vaginal commensals and indicated that it does not modify the vaginal microbiome (Husain et al., 2020). It is clear that the vaginal microbiome impacts mom pregnancy health and that of the offspring both in infectious and noninfectious setting although, the mechanism are less understood. To address this gap, the NIH and the Global Alliance to Prevent Prematurity and Stillbirth (GAPPS) are collaboratively working on a Multi‐Omic Microbiome Study‐Pregnancy Initiative (MOMS‐PI). The initiative aims to investigate the impact of pregnancy on the maternal microbiome; how the microbiome affects maternal host response and the influence of the microbiome on neonatal health. The exact contribution of the vaginal microbiome to the etiology of preeclampsia remains to be evaluated.

## 7 PLACENTAL MICROBIOME

The mere existence of a placental microbiome is a subject of great controversy. There are reports to support that the placenta has its own distinct microbiome while others argue the opposite. Before the placental microbiome was evaluated, early studies showed the existence of specific pro‐inflammatory bacteria such as *Helicobacter pylori* in placentas from preeclamptic women (Ponzetto et al., 2006) and the presence of intracellular bacteria in the human maternal‐fetal interface, predominantly in fetal extravillous trophoblasts (Cao & Mysorekar, 2014). There is also evidence of specific pro‐inflammatory bacteria such as *Helicobacter pylori* in placentas from preeclamptic women. Other reports characterized the unique placental microbiome to be composed of nonpathogenic microbiota, independent of the mode of delivery (Aagaard et al., 2014; Doyle et al., 2014). An interesting study in rodents also reported the presence of placental microbiome but suggested that it potentially originates from another cavity like the mouth through translocation (Fardini et al., 2010).

On the other hand, others have reported the lack of detection of any placental microbiota both in placentas from normal pregnancies and preterm delivery placentas as well as in rodents (Goffau et al., 2019; Leiby et al., 2018; Theis et al., 2020). They speculate that the placental microbiome reported by others is due to contamination during delivery and from reagents (Goffau et al., 2019). Given the substantial role that the placenta plays in the pathophysiology of preeclampsia, its microbiome has also been examined in the development of the disease. Bacteria were detected in placentas from hypertensive pregnancies and pregnancies associated with low birth weight (Onderdonk et al., 2008; Swati et al., 2012). The presence of bacteria in placentas from preeclamptic women has also been described, although, the species found were reported to normally be commensals of the oral, gut, or respiratory tract microbiomes (Amarasekara et al., 2015; Barak et al., 2007). So far, only one study demonstrated both the presence of microbial organisms that were of the placenta and others distinguished to be from reagent contamination. These results emphasize the need for more, better‐controlled studies before dismissing the potential role of the placental microbiome in pregnancy disorders such as preeclampsia (Leon et al., 2018).

Scientific development in the last decade demonstrates that the microbiome plays a role in human health and disease, especially gut bacteriome. More studies characterizing not only the microbial changes in normal pregnancy but also the associated metabolites modulating healthy adaptations are warranted. Although, this discussion focused on the effect of the microbiome in pregnancy outcomes, it is important to acknowledge that related factors such as hormones, diet, and medications can also affect the microbiome. Furthermore, with controversies in low‐density microbial habitats like the placenta, more studies are needed that are well controlled to not only establish taxonomic composition but also functional pathways and signaling gut‐derived metabolites.

## 8 MICROBIOME DYSREGULATION IN PREECLAMPSIA: NEW‐ONSET VERSUS DORMANT MICROBIOME

It is not known whether the dysbiosis associated with preeclampsia is a new onset feature or whether pregnancy acts as a “second hit” to trigger the activation of a previously dormant microbiome (Kell & Kenny, 2016). Although, the theory is yet to be tested, it is certainly interesting considering that microbes and dysbiosis exist in other tissues and conditions outside of pregnancy. For instance, a previous study detected “live gut bacteria” in the circulation of diabetic patients (Sato et al., 2014). The uterine microbiome has also been characterized in non‐pregnant women (Verstraelen et al., 2016). Maternal infections are known to play a role in adverse pregnancy outcomes (Cassinji et al., 2013; Jarjoura et al., 2005; Madianos et al., 2001), and intervening early and treating underlying conditions such as administration of antibiotics to treat infections improves pregnancy outcomes (Herrera et al., 2001; Menezes et al., 2009). These outcomes include extending the gestation period and/or reducing preterm birth. As we previously discussed, other risk factors such as chronic hypertension, diabetes, and obesity are associated with a disrupted microbiome. The contribution of a preexisting dysbiosis in developing preeclampsia and the mechanisms involved remain to be elucidated.

## 9 THE PATHOPHYSIOLOGY OF PREECLAMPSIA AND MECHANISMS OF THE MICROBIOME: WHAT IS THE CONNECTION?

Successful placentation, mediated by mechanisms such as trophoblast invasion, plays a critical role in normal pregnancy, and abnormalities in the process play a role in the pathophysiology of preeclampsia (Armaly et al., 2018). The placenta may harbor pathogenic microbes and potentially have its own microbiome. To fight placental infection, decidual natural killer cells protect the placenta through cytolytic effectors such as perforin and granzymes to selectively kill pathogens, sparing normal host cells thus protecting the placenta from intracellular infections (Crespo et al., 2020) such as *Listeria monocytogenes* that have been previously detected in placental trophoblasts (Cao & Mysorekar, 2014). However, this mechanism is said to be protective only when it occurs in earlier stages of pregnancy. Continued heightened cytotoxic activity of decidual natural killer cells during pregnancy can be harmful and has been implicated in the development of preeclampsia (Travis et al., 2020). This suggests that placental infections in late pregnancy stages may increase the risk of developing adverse pregnancy outcomes.

Research exploring the mechanisms by which the microbiome regulates hypertensive disorders have predominantly focused on the gut microbiome. As such, this discussion will explore some of these mechanisms and how they fit into our current understanding of the pathophysiology of preeclampsia. Normally, microbes in the gut exist in a symbiotic relationship with the host where they play a role in maintaining physiological homeostasis. Numerous disease states are associated with an imbalance in the microbiota accompanied by a dysfunction in the integrity of the gut barrier. As a result, microbial communities and derived signaling metabolites can enter host circulation and activate a variety of pathways, contributing to diseases development and progression (Tang et al., 2017). In mice, microbial imbalance in the gut was associated with colon pathologies evaluated through markers such as mucosal inflammatory cell infiltration, hyperplasia, and goblet cell number (Sun et al., 2018). Antibiotic treatment in rhesus monkeys‐induced aberrant small intestinal structure characterized by denatured and necrotic epithelial cells of the intestinal mucosa (Li, Li, et al., 2020). Adult spontaneously hypertensive rats have a leaky intestinal barrier and decreased expression of tight junction proteins like occludin, tight junction protein 1, and claudin 4 in the small intestine compared to normotensive controls (Santisteban et al., 2017). Although, less is known about the intestinal mucosa morphology in humans, an imbalanced gut microbiome is associated with changes in proteins that regulate the gut barrier. For example, hypertensive patients had increased expression of zonulin which strongly correlated with blood pressure (Kim et al., 2018). The changes in gut wall integrity in preeclampsia are not understood, but we can gain insight from changes that occur in other pregnancy disorders. Interestingly, maternal high‐fat diet in mice shifts the structure of the gut microbiome and results in impaired gut integrity as well as placental blood vessel immaturity and hypoxia (Gohir et al., 2019). Maternal obesity induces intestinal oxidative stress and impaired gut barrier function in the offspring (Xue et al., 2014). Maternal malnutrition also alters maternal gut homeostasis and fetal gut wall permeability further implicating a developmental programming component (Srugo et al., 2019).

Bacteria make several metabolites through fermentation of nutrients in the gut which can enter circulation and exert physiological effects (Al Khodor et al., 2017). More importantly, because bacterial taxa associated with diseases widely vary among studies, understanding the metabolites and the pathways they activate holds tremendous translational implications. This is important because the detection of bacterial composition in environments does not indicate their function. Gut‐derived LPS, which activates Toll‐like receptors to increase pro‐inflammation, is increased both in hypertensive and preeclamptic patients together with a disrupted gut microbiome (Gohir et al., 2019; Kim et al., 2018; Lv et al., 2019; Wang et al., 2019). LPS is thought to play a role in the pathogenesis of preeclampsia and is used to generate rodent models of the disease (Fan et al., 2019). Probiotics treatment that decreased plasma LPS levels also attenuated hypertension and prevented endothelial dysfunction (Robles‐Vera et al., 2020). Preeclampsia is associated with increased circulating TMAO levels and probiotics treatment prevents blood pressure elevation in pregnant rats on a high‐fat diet (Hsu et al., 2019; Wang et al., 2019). Probiotics treatment improved vascular oxidative stress and decreased pro‐inflammatory in rats with antihypertensive effects suggested to be mediated by nitric oxide pathways (Robles‐Vera et al., 2018). Cardiovascular dysfunction in pregnancy plays a role in preeclampsia, but the role of microbiome in modulating vascular or hemodynamic changes in pregnancy are not known. We speculate that the microbiome may contribute by modulating vascular function via nitric oxide, immune mechanisms such as interleukin‐17 and vasoactive metabolites such as TMAO (Kalafat & Thilaganathan, 2017; Karbach et al., 2016; Restini et al., 2021).

Numerous studies have indicated an association between decreased levels of short‐chain fatty acids (SCFAs) like acetate, propionate, and butyrate and hypertension (Hu et al., 2019; Kim et al., 2018; Kim, Rigatto, et al., 2020) by activating G‐protein‐coupled receptors (GPR) 41, 43, and 109A (Li, Chen, et al., 2020; Natarajan et al., 2016). Most of these metabolites induce changes in host physiology by mediating immune mechanisms. For example, butyrate regulates differentiation of Th17 and Th1 cells (Chen, Sun, et al., 2019). Th1 cells mediate interleukin‐10 production through G‐protein‐coupled receptor 43 (Sun et al., 2018). The adequate regulation of immune mechanisms is particularly important in pregnancy because not only is its impairment associated with disorders like preeclampsia (Geldenhuys et al., 2018; LaMarca et al., 2016), but also the state of maternal microbiota also programs early innate immune development in the offspring (De Agüero et al., 2016).

## 10 PRE‐CLINICAL MODELS OF PREECLAMPSIA IN MICROBIOME RESEARCH

The microbiota is influenced by many factors and is highly dynamic even in healthy individuals (Lloyd‐Price et al., 2017). To account for the potential confounders and avoid variation in microbiome research findings, there are guidelines and considerations available to increase rigor and reproducibility especially in studying hypertensive disorders (Marques et al., 2019). The design and reporting on factors like diet in human studies assessing the microbiome changes in pregnancy have been inconsistent perhaps due to their longitudinal nature.

Spontaneous models of preeclampsia provide a preclinical alternative in establishing the changes in the microbiome that are specific to pregnancy and their timeline in a controlled experimental setting. Female BPH/5 mice are borderline hypertensive as virgins and develop gestational hypertension, renal disease, and adverse fetal outcomes among other features that are similar to those seen in preeclampsia (Davisson et al., 2002). The Dahl Salt‐sensitive (S) rat that is normally chronically hypertensive develops a superimposed preeclampsia‐like phenotype exhibiting many characteristics of the disease including hypertension, proteinuria, and increased pro‐inflammatory cytokines (Gillis et al., 2015). Using this model, we recently showed that gut microbiome changes vary by gestation stage, and that chronic hypertension that precedes PE in the model may hinder the necessary gut microbial remodeling during pregnancy. We report impaired gut microbiome during pregnancy further validating the use of this model in microbiome preeclampsia preclinical studies (Ishimwe et al., 2021). BPH/5 mice, Dahl S rats, and other spontaneous models (Rhoads et al., 2017; Sharkey et al., 2001) provide invaluable tools to understand microbiome alterations at each pregnancy stage; test gut‐targeted therapies in treating preeclampsia, and explore the effect of the maternal microbiome on offspring health outcomes.

Current reports implicating the role of the microbiota in preeclampsia are predominantly association‐based evidence. Assessing longitudinal changes in the microbiome composition of spontaneous preclinical models may identify specific bacteria species or strains that are differentially changed compared to normal pregnant controls. To directly investigate a causative role of one or multiple bacteria on host disease phenotypes such as preeclampsia, germ‐free, or gnotobiotic animals will be indispensable tools for this purpose (Basic & Bleich, 2019). The confirmed “disease‐causing” species might provide an alternative, affordable, and non‐invasive biomarker approach for preeclampsia. Also, identifying these specific bacteria will guide in further assessing the mechanisms involved for instance through targeted metabolomics. Particularly, studying the microbial communities such as that of the placenta in these animals would be useful in differentiating the actual microbiome from potential detection of contamination as well as specifics in the microbiome shift that occur in normal versus preeclamptic pregnancies. However, germ‐free animals are limited in their usefulness in studying immune‐related mechanisms because they have developmental defects in their gut wall structure and in immune responses (Round & Mazmanian, 2009).

There is also room for other models of induced preeclampsia‐like phenotype by reduced uteroplacental perfusion (RUPP), promoting anti‐angiogenesis, immune activation, and others in microbiome studies (Albers et al., 2019; Hasan et al., 2015; Hayakawa et al., 2000; Li et al., 2012; Lu et al., 2007; Zhou et al., 2008). The interventions to induce the phenotype in these models are typically carried out in the mid‐pregnancy stage. Therefore, we speculate that these models may not be best to use in characterizing changes in host‐microbial communities specific to preeclampsia because the surgical procedures and the pharmacological agents would confound the microbiome results (Marques et al., 2019). The main limitation lies in that the period between mid‐pregnancy and birth of the pups does not leave enough time for the microbiome to reset. LPS or endotoxin is found on the membrane of gram‐negative bacteria. Plasma LPS is increased in preeclamptic women along with gut imbalance (Wang et al., 2019). Administration of LPS in rodents also results in a preeclampsia‐like phenotype by mediating systemic inflammation (Xue et al., 2015). Although, not a suitable model for characterizing pregnancy‐specific microbiome changes,the LPS model of preeclampsia is a good example of direct effects of the pharmacological agent on the gut microbiome. Collectively, this category of preclinical models of preeclampsia has long been useful in testing potential treatments (Li, Wang, et al., 2019; Sekimoto et al., 2020; Zhao et al., 2018). Thus, they should also be employed in assessing the efficacy of gut‐targeted therapies on both maternal and fetal outcomes during pregnancy and long‐term. For example, inhibiting TMAO production by 3,3‐Dimethyl‐1‐butanol ameliorated endothelial function and hypertension in the RUPP model (Chen et al., 2019).

## 11 MATERNAL MICROBIOME: IMPLICATIONS ON OFFSPRING HEALTH

It is established that maternal and uteroplacental environments greatly influence the health outcomes of the offspring long‐term through developmental programming mechanisms (Alexander, 2006). Preeclampsia is associated with fetal growth restriction and greater risks for developing cardiovascular disorders in the offspring (Jim & Karumanchi, 2017; Sharkey et al., 2001; Turbeville & Sasser, 2020). Although, the exact contribution of the microbiome on adverse fetal outcomes associated with preeclampsia is not completely understood, there are plenty of studies highlighting the impact of the maternal microbiome on offspring health (Moore & Townsend, 2019; Turroni et al., 2020). Evidence suggests that alterations in the maternal microbiome also impact infant microbiome, and prenatal interventions protect offspring from developing cardiovascular diseases like hypertension (Dotterud et al., 2015; Hsu et al., 2018; Kim, Allen, et al., 2020; Sohn & Underwood, 2017; Torres et al., 2020). In addition to the incomplete understanding of mechanisms by which maternal microbiome affects offspring health, the start and mode of fetal exposure to the maternal microbiome are controversial. The womb was long thought to be sterile and there is still evidence supporting this hypothesis (Malmuthuge & Griebel, 2018; Theis et al., 2020). Au contraire, emerging reports suggest that fetus exposure to microbes may start in utero. This theory is substantiated by the reported presence of microbes in tissues such as the placenta, amniotic fluid, and the meconium (Combs et al., 2015; Doyle et al., 2014; Moore & Townsend, 2019; Walker et al., 2017). Bacteria in the meconium resemble those found in the amniotic fluid and are more prevalent in preterm infants (Ardissone et al., 2014).

The currently accepted dogma is that the initial fetal exposure to the microbes occurs at birth via maternal vaginal microbiome or maternal skin microbiome if the baby is born by caesarian section (Ferretti et al., 2018; Maqsood et al., 2019). Other neonatal microbial exposure sources include maternal intrapartum antibiotics, postnatal stay in the hospital, breastfeeding (immediate, partial, or exclusive), and postnatal antibiotics. Health outcomes in offspring are suggested to vary depending on the mode by which they were delivered. Vaginal birth, breastfeeding, and early childhood exposure to various microbes are some of the ways reported to set a child up for a more symbiotic and protective microbiome (Azevedo et al., 2020; Funkhouser & Bordenstein, 2013; Schei et al., 2017; Shao et al., 2019; Van Nimwegen et al., 2011). The effect of the maternal gut microbiome on the offspring persist longer than those from exposure to vaginal and skin microbiome (Ferretti et al., 2018). However, that hypothesis assumes that the maternal gut or vaginal microbiome that the fetus was exposed to was healthy. Whether the microbiome effects are dependent on maternal health status and the state the gut microbiome warrants further exploration (Koren et al., 2012; Yao et al., 2020).

## 12 MATERNAL MICROBIOME, FETAL PROGRAMMING AND FUTURE HEALTH OUTCOME

It is established by the developmental origins of health and disease (DOHaD) hypothesis that exposure to unfavorable environments during critical periods of development and growth have long‐term consequences on health (Barker, 2007). Developmental programming has been implicated in numerous diseases including metabolic disorders and hypertension (Fall & Kumaran, 2019; Hsu & Tain, 2020; Turbeville & Sasser, 2020). Although, there are still many open‐ended questions on the role of the maternal microbiome and transmission to their offspring, it is evident that the microbiome plays an immense role in shaping numerous physiological systems. The shape of the microbiome in a person's first 1000 days, spanning the day of conception through the first 2 years of life, is suggested to be a crucial player in future health status further emphasizing the importance of adequate development programming (Robertson et al., 2019). Preterm infants born before 33 weeks of gestational age demonstrate impaired bacterial colonization that may be related to insufficient perinatal gut maturation (Butel et al., 2007). Limited exposure to factors that deleteriously affect the gut microbiome during early life, cause long‐term immunometabolic consequences even after the gut biodiversity recovers (Cox et al., 2014).

**FIGURE 1:**
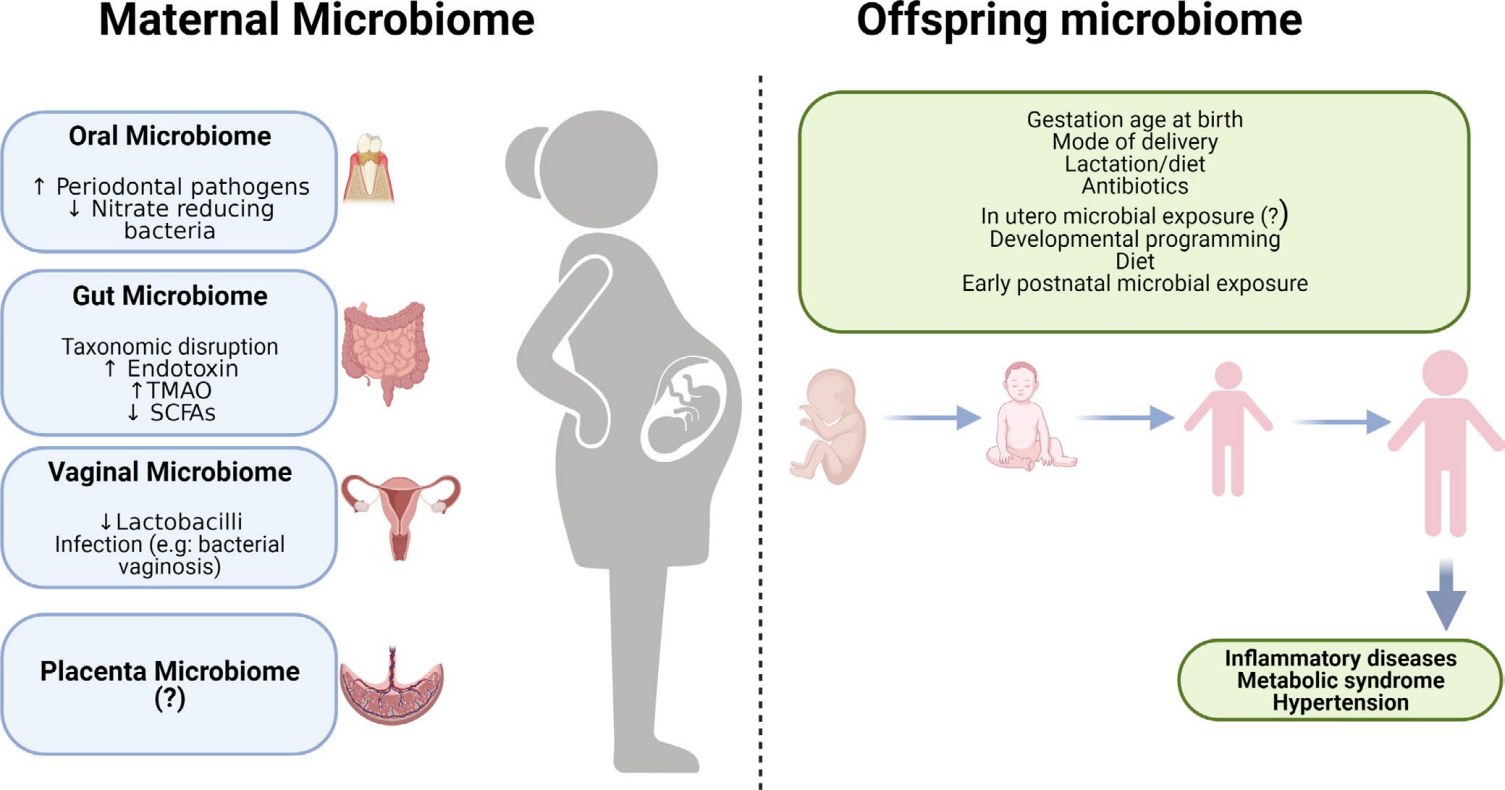
Maternal microbiome environments that contribute to preeclampsia and developmental origins of health. Dysbiosis in pregnancy is associated with increased pathogenic microbes and metabolites that cause hypertension and adverse fetal outcomes. Exposure to a disrupted maternal microbiome in utero and during early life impacts future health outcomes in the offspring. This figure was created with BioRender.com

The maternal microbiome can also influence fetal health by altering fetal gut health and modulating immune mechanisms to protect from or contribute to disease development (De Agüero et al., 2016; Koch et al., 2016; Srugo et al., 2019). Mice born from dams treated with antibiotics during pregnancy display impairment in adaptive immunity (Nyangahu et al., 2018). Similarly, antibiotic exposure during pregnancy is associated with increased risk for asthma is children (Zhao et al., 2015). When the maternal microbiome is imbalanced due to being overweight during pregnancy, vaginal birth is associated with disruption in infants gut microbiome that is not seen in infants born from overweight mothers delivered via the cesarean route (Singh et al., 2020). These findings suggest that if the maternal microbiome is pathogenic, minimizing fetal expose to it may be protective. On the other hand, the hygiene hypothesis suggests that early childhood exposure to microbes contributes to the development of the immune system and is associated with better health outcomes in life (Alexandre‐Silva et al., 2018). Studies have demonstrated that children who grow up on in rural areas and on farms have a resilient gut and are less prone to disease (Ege, 2017; Mellander et al., 1985). Other diseases that have been associated with developmental programming defects in the microbiome include allergy, psychiatric disorders, inflammatory bowel disease, and hypertension (Codagnone et al., 2019; Hsu et al., 2019; Littman & Pamer, 2011; Vuillermin et al., 2017). We have summarized the role of the microbiome in preeclampsia and how it may affect their offspring in Figure 1.

## 13 THE THERAPEUTIC POTENTIAL OF THE MICROBIOME

Preeclampsia is a devastating disease with a poorly understood etiology and no approved therapies to treat it. It also increases the risk of developing the cardiovascular disease for both mom and offspring. Therefore, elucidating the pathophysiology of preeclampsia and finding treatments will improve pregnancy outcomes and long‐term health status in mothers and their offspring. The microbiome undoubtedly plays a crucial role in regulating normal host physiology. It is important to note most microbiome studies in preeclampsia have thus far reported association with the disease but not causation. Our review of the literature so far has highlighted ways in which alterations in the host and microbial relationship contributes to disease. Understanding this relationship can be harnessed into developing novel therapeutic strategies. Gut‐targeted therapies have been extensively shown to improve risk factors and characteristics of preeclampsia including obesity, diabetes, hypertension, and inflammation (Frei et al., 2015; Sáez‐Lara et al., 2016). Below we review current treatment approaches that target the microbiome, the challenges, and potential future directions.

## 14 PREBIOTICS, PROBIOTICS, AND SYNBIOTICS

Diet is well known to affect gut health by promoting the survival of certain commensals, starving other commensals, and through their effects on gut wall barrier integrity. Prebiotics, such as cereals and artificial sources like inulin, are nondigestible compounds that improve the intestinal microbiota composition and activity. Probiotics are live microorganisms that confer a health benefit on the host when administered in adequate amounts (Hill et al., 2014). Synbiotics are a combination of prebiotics and probiotics (Patel & Dupont, 2015). Probiotics are typically one or a combination of microbial strains such as *Lactobacillus*, *Bifidobacterium*, *Lactococcus*, *Streptococcus*, and *Enterococcu*s. The Food and Drug Administration in the United States regulates microorganisms based on their Generally Regarded As Safe (GRAS) status (Markowiak & Ślizewska, 2017).

Probiotics are generally considered to be safe during pregnancy and to be potentially beneficial in preeclampsia (Sohn & Underwood, 2017). Their mechanisms of actions and therefore, efficacy as therapeutics depend on the strains in the formulation. This may explain why a lot of probiotics are unsuccessful as treatments. For instance, probiotics made of Lactobacillus rhamnosus and Bifidobacterium animalis strains did not prevent gestational diabetes during pregnancy in the SPRING trial or improve maternal outcomes in the Healthy Mums and Babies (HUMBA) trial (Callaway et al., 2019; Okesene‐Gafa et al., 2019). A second and/ or alternative explanation may lie in the time of intervention initiation. The Norwegian Mother and Child Cohort Study demonstrated that probiotic milk intake lowers the risk of developing preeclampsia, an effect not observed when the probiotic was taken before or early in pregnancy (Nordqvist et al., 2018). A better understanding of the specific microbial alterations in each disease like preeclampsia will provide insights into choosing the right intervention instead of considering prebiotics, probiotics, and synbiotics as one size fits all.

## 15 FIBER

Fiber is another gut‐targeted therapy that modulates many pathways known to play a role in blood pressure regulation (Holscher, 2017; Marques et al., 2017). Dietary fiber refers to nondigestible carbohydrates that are intrinsic and intact in plants. Functional fiber comprises isolated and manufactured, nondigestible carbohydrates that have beneficial physiological effects. The definition of fiber varies around the world, but the consensus is that all prebiotics are fibers but not all fibers are prebiotics (Howlett et al., 2010; Slavin, 2013). Fermentable fibers such as inulin and fructooligosaccharides induce the growth and activity of beneficial microbes (Poeker et al., 2018). Fiber consumption is associated with elevated blood SCFAs concentration, and improves gut health by attenuating intestinal inflammation (Foye et al., 2012; Koh et al., 2016; Whelan et al., 2005). Pregnancy women are recommended to consume 29 g/day of fiber (Dahl & Stewart, 2015). Dietary is a strong modifiable risk factor for cardiovascular disease. Dietary counseling to encourage high fiber intake in pregnant women should be assessed further as a potential preventative and management strategy in preeclampsia. The idea is strengthened by evidence from animal and human studies indicating that perinatal fiber extends benefits to the offspring. These include improved growth rate, intestinal barrier integrity, and decreased susceptibility to allergy (Cheng et al., 2018; Pretorius et al., 2019).

## 16 GUT‐DERIVED METABOLITES

Microbes in the gut make a wide range of metabolites including bile acids, uremic toxins, TMAO, LPS, and SCFAs. Although these metabolites like short‐chain fatty acids can be intracellular effector molecules of prebiotics, prebiotics, and synbiotics, they can also affect host physiology when administered independently. The SCFAs acetate and propionate have antihypertensive effects, improve endothelial function, and attenuate inflammation (Bartolomaeus et al., 2019; Ganesh et al., 2018). Therapeutic effects of butyrate have been extensively studied and shown to be beneficial in numerous diseases hypertension and kidney disease (Gonzalez et al., 2019; Wang et al., 2017; Zhou et al., 2017). Butyrate modules gut wall integrity by promoting colonic mucus production (protective) and increasing tight junction proteins, therefore, limiting the release of toxic metabolites into circulation (Gonzalez et al., 2019). Butyrate levels inversely correlate with pro‐inflammatory markers and nitric oxide levels in humans, the latter effect not seen in other SCFAs (Juanola et al., 2019). The effects conferred by SCFAs such as lowering blood pressure, attenuating inflammation, and improving endothelial function effects conferred by SCFAs would be beneficial in preeclampsia, and therefore, these compounds should be explored as potential therapeutics. Additionally, developing agonists and antagonists for protective and harmful pathways respectively may reveal new therapies for diseases such as preeclampsia. Some of these include TLR4, GPR41, GPR43, and GPR109A signaling pathways.

## 17 CONCLUSION

More studies are warranted to determine the role of gut‐targeted therapies for the treatment of preeclampsia. Also, the safety of these treatments in pregnancy needs to be evaluated. Furthermore, while these interventions are suggested to target the gut, their current design is not specific to the gut. Future advancement in the therapeutic development will be crucial in increasing their safety, viability past the stomach pH. Specificity also needs to be improved especially for conditions like preeclampsia to ensure the fetus's safety and avoid off‐target effects. The progress in the microbiome field is promising as a target for managing and treating diseases. Elucidating disease‐specific microbial aberration will improve the selection of potential therapeutic. By combining metagenomics, metabolomics, and proteomics technologies, we will likely continue learning of new metabolites and receptors mediating microbial effects on the host. Upon review, the microbiome holds promising therapeutic implications for the treatment of preeclampsia and potentially reprogramming‐related fetal health consequences.

## CONFLICT OF INTEREST

No conflicts of interest, financial or otherwise, are declared by the authors.

## AUTHOR CONTRIBUTIONS

J.A.I prepared figures; J.A.I drafted manuscript; J.A.I edited and revised manuscript; J.A.I approved final version of manuscript.
